# Religion is Secularised Tradition: Jewish and Muslim Circumcisions in Germany

**DOI:** 10.1093/ojls/gqaa028

**Published:** 2020-12-14

**Authors:** Lena Salaymeh, Shai Lavi

**Affiliations:** 1 Professor of Law, Tel Aviv University and Director, Van Leer Jerusalem Institute; 2 British Academy Global Professor, University of Oxford and Affiliate, Max Planck Institute for Comparative and Private International Law. Email: slena@tauex.tau.ac.il

**Keywords:** religion, secularism, tradition, circumcision, Germany, Jews, Muslims

## Abstract

This article demonstrates that the legal reasoning dominant in modern states secularises traditions by converting them into ‘religions’. Using a case study on Germany’s recent regulation of male circumcision, we illustrate that religions have (at least) three dimensions: religiosity (private belief, individual right and autonomous choice); religious law (a divinely ordained legal code); and religious groups (public threat). When states restrict traditions within these three dimensions, they construct ‘religions’ within a secularisation triangle. Our theoretical model of a secularisation triangle illuminates that, in many Western states, there is a three-way relationship between a post-Christian state and both its Jewish and Muslim minorities. Our two theoretical proposals—the secularisation triangle and the trilateral relationship—contribute to a re-examination of religious freedom from the perspective of minority traditions and minority communities.

## 1. Introduction

Recent scholarship has offered new ways of understanding secularism from a non-Eurocentric perspective; secularism is no longer perceived in a simplified way as the ‘separation of state from religion’.^1^ Instead, secularism is widely recognised as shaping modern governance, even in states that may be perceived as ‘non-secular’. There are, of course, variations in European, North American and other forms of secularism. Secular states may have an established religion or no established religion, or be overtly anti-religion.^2^ While it is common to distinguish between ‘less’ and ‘more’ secular states—based on a state’s official treatment of religion or a state’s demographics—such distinctions can be misleading.^3^ Whether they promote assimilation or multiculturalism, or prohibit or recognise religious institutions, modern states construct ‘religion’ and regulate ‘religious freedom’ in similar ways.^4^ When we shift away from a Eurocentric perspective, secularism’s particularities become less discernible and its broad patterns become clearer. Focusing on abstract dynamics, this article demonstrates how modern states *secularise* traditions by constraining them within the category of religion. Religion is not a transhistorical phenomenon, but rather a modern category that is produced by secularism.

**Figure 1:**
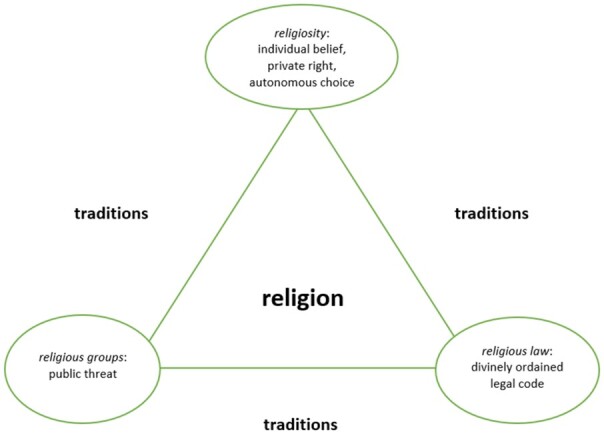
Secularisation triangle (a schematic diagram).

Throughout this article, the term ‘the state’ refers to modern secular states; we recognise that states do not have agency and that secular states are diverse.^5^ Nevertheless, we propose that the legal reasoning dominant in modern states secularises traditions by converting them into religions.^6^ A tradition is a changing and multi-vocal array of ideas and practices shared by groups over time.^7^ We concentrate on Western states, which generally synthesise Protestant Christian traditions and modern, fluctuating interests of the state; accordingly, secular states cannot be classified as ‘basically Christian’ or ‘totally nonreligious’. We illustrate that many religions have (at least) three dimensions: religiosity (private belief, individual right and autonomous choice); religious law (a divinely ordained legal code); and religious groups (public threat). When states restrict traditions within these three dimensions, they construct ‘religions’ within a secular framework. We refer to these three dimensions as a secularisation triangle (see Figure 1), which we will elaborate below. A state may not impose these three dimensions simultaneously and there may be more dimensions to the secularisation of traditions. We focus on these three dimensions of a state’s secularisation of traditions because states use them as evidence that they are *accommodating* minority communities. Rather than accommodating minorities, states secularise the traditions of minorities.^8^ Modern state actors often claim that neutral principles or generally applicable rules are the basis of refusing to protect certain practices.^9^ As we elaborate in this article, these seemingly neutral notions or purported government interests are subjective and biased.

Our proposal of a secularisation triangle is a theoretical model that is neither empirical nor historical; instead, the secularisation triangle is a representation of broad patterns in secular legal reasoning that are detectable in many secular states. The secularisation triangle is based on numerous case studies that we taught together over several years. Although we built our approach on several cases from a wide range of states, in this article, our prime example comes from the German regulation of circumcision. The relevance of our argument to other states—particularly those beyond Europe—requires further study; however, we anticipate that analogous dynamics animate the place of religion in other jurisdictions.^10^ Our objective in this article is to invite other scholars to test our models in their own areas of specialisation.

In addition to abstract dynamics, our secularisation triangle illuminates that in the particular situations of many Western states, state regulation of religion is seldom simply a question of the relationship of one state to one minority. In many situations, there is a three-way relationship between a post-Christian state and both its Jewish and Muslim minorities. We present two interrelated arguments about this trilateral relationship by building upon critical theories of secularism, which have contributed to ‘law and religion’ scholarship (particularly in the area of religious freedom) in illuminating ways.^11^ First, states modify historical Christian criticisms of Judaism in their construction of religion. Accordingly, some contemporary secular legal reasoning modifies historical Christian polemics against Judaism. Secondly, and relatedly, states evaluate Islamic practices by comparing them to Jewish practices.^12^ Specifically, states often adopt a Christian historical perspective that categorises traditions in a linear, chronological hierarchy, such that Judaism, the ‘older’ tradition, is elevated above Islam, the ‘younger’ tradition.^13^ Since this Christian-influenced perspective perceives Judaism as ‘original’ and Islam as ‘derivative’, states frequently evaluate Muslim practices in relation to Jewish practices. Although we will not elaborate the point fully in this article, it is important to recognise that the state’s secularisation triangle (religiosity, religious law and religious group) has differential consequences for Jews and Muslims. Judaism and Islam are not interchangeable traditions; because Jewish and Islamic traditions are distinct, their secularisation as religiosity, religious law and religious group has dissimilar results. Consequently, although states discriminate against both Jews and Muslims, there is an additional level of discrimination against Muslims.^14^

Our case study is Germany’s recent regulation of male circumcision, which has been the subject of debate and critique in many parts of Europe, including Iceland,^15^ Norway,^16^ Denmark^17^ and the Netherlands.^18^ Despite the significance of Catholicism in Germany, German secularism combines Protestant Christian ideas and modern, fluctuating interests of the German state.^19^ The German state portrays circumcision as a matter of religiosity, regulates circumcision with reference to religious law and distinguishes circumcision based on the state’s classification of its practitioners as a religious group. In addition to demonstrating the secularisation triangle in Germany, this case study elucidates the trilateral relationship between the post-Christian secular state, its Jewish minority and its Muslim minority. First, German law uses modified forms of historical Christian polemics against Jewish circumcision in evaluating the practices of both Jews and Muslims. Secondly, German law evaluates Muslim circumcision in relation to Jewish circumcision.^20^ There are dimensions of this case study that may resonate in many secular states and there are other dimensions that may resonate only in Western or Christian-majority states.

This article proceeds as follows. Section 2 reviews and comments on the existing scholarly literature in critical theories of secularism, focusing on how we contribute to and expand recent scholarship on religious freedom. Section 3 identifies the secularisation triangle in our German circumcision case study and explores the trilateral relationship between the German state and its Jewish and Muslim minorities. Section 4 examines a prior historical controversy about circumcision in Germany that exemplifies how states secularise traditions by converting them into religions. Our two theoretical proposals—the secularisation triangle and the trilateral relationship—contribute to a re-examination of religious freedom from the perspective of minority traditions and minority communities.

## 2. Building on Critical Theories of Secularism

Our objective is to refine the existing legal-analytical tools for evaluating *how* states discriminate against minorities under the doctrine of religious freedom. The particular legal-analytical tools we develop are modifications of a genealogical approach to the study of secularism. Critical theories of secularism demonstrate that secularism is not a neutral or universalist ideology because its historical beginnings in the Protestant Christian tradition moulded its approach to ‘religion’.^21^ While we build upon critical secularism studies, we also recognise some common concerns and possible limitations of this approach. Sarah Shortall highlights three potential pitfalls: that it is essentialist (presuming that secularism is essentially Christian), homogenising (ignoring dissimilarities within Christian denominations) and unidirectional (emphasising only how Christianity influenced secularism and not vice versa).^22^ We concur with and recognise the importance of Shortall’s observations, as well as the geographic diversity and temporal changes in secularism. We do not conflate secularism with Protestant Christianity; as previously noted, we acknowledge that secularism combines Protestant Christian ideas and emerging interests of the modern state. We appreciate the contextual particularities of secularism, particularly how secular ideology interacts with racism and nationalism.^23^ We also recognise that there are multiple and diverse Christian denominations, and we have attempted to be detailed in our discussions of Christian traditions. Nevertheless, as non-specialists in Christian theology or the history of Christianity, our objective is to clarify how particular secular ideas contrast with Jewish and Islamic traditions, rather than precisely where or how specific secular ideas emerge from distinctive Christian traditions. Secularism, despite its local and historical variations, is an ideology and array of practices that may be analysed for general patterns that are more consistent with Christian traditions than with Jewish or Islamic ones.

In this section, we outline how critical secularism studies challenges many conventional assumptions within the field of ‘law and religion’. Most importantly, as will be elaborated below, ‘law and religion’ scholarship commonly presumes that secularism separates religion from state governance. In contrast, critical secularism studies shows the impossibility of disentangling religion from state governance. This crucial distinction between these two general approaches stems from their dissimilar understanding of ‘religion’. Critical secularism studies emphasises that secularism (influenced by the Protestant Christian tradition) defines religion. Thus, religion is not a transhistorical phenomenon. Indeed, religion may be defined as non-secular.^24^ In general, scholars outside critical secularism studies perceive religion as a transhistorical phenomenon, even if they acknowledge the difficulty of defining religion. These two distinct conceptions of religion have numerous implications within the field of law and religion. Cultivating the critical secularism perspective, we illustrate that secular notions of religion are incongruent with Jewish and Islamic traditions.

The question of how law should or should not define religion has animated much law and religion scholarship.^25^ Many scholars have noted that Western secular states define religion according to Protestant Christian assumptions.^26^ Some scholars have suggested that it is impossible for law to define religion.^27^ Other scholars propose that an explicit or comprehensive legal definition of religion is unnecessary. For instance, the Research Division of the European Court of Human Rights (ECtHR) claims that failure to define religion in article 9 of the European Convention on Human Rights (ECHR) is ‘logical, because such a definition would have to be both flexible enough to embrace the whole range of religions worldwide … and specific enough to be applicable to individual cases’.^28^ Similarly, Daniel Philpott and Timothy Shah acknowledge the difficulty of defining religion, but maintain that ‘religion represents a genuine, transcultural, if elusive human phenomenon’ that is not specific to the Protestant Christian tradition in the West.^29^ Because these abstract debates often begin from the perspective of secularism, they underestimate non-Christian traditions. From the perspective of Jewish and Islamic traditions, secular states define religion by fusing Protestant Christian ideas with the state’s practices.

The definitional conundrum surrounding religion has significant implications for the legal doctrine of religious freedom. Protestant Christian conceptions are evident, for instance, in how the legal principle of ‘freedom of religion’ both identifies ‘legitimate’ religious practices and polices the border between religion and non-religion. In turn, the doctrine of ‘freedom of religion’ participates in the secular process of defining religion as apolitical. The Research Division of the ECtHR elaborates this dynamic, noting that Article 9 of the ECHR 'protects a person’s private sphere of conscience but not necessarily any public conduct inspired by that conscience’.^30^ Saba Mahmood and Peter Danchin explain that it is the ‘distinction between *forum internum* and *forum externum* that essentially allows the state simultaneously to uphold the immunity and sanctity of religious belief *even* as it regulates the manifestation of these beliefs … this antinomy is internal to the conceptual architecture of the right [to freedom of belief] itself’ .^31^ Simply put, the secular state offers individuals freedom of belief, but not the freedom to act publicly based on those beliefs.

Accordingly, in recent years, critical secularism studies has called attention to the contradictions and complexities of ‘religious freedom’.^32^ Danchin, Winnifred F Sullivan, Mahmood and Elizabeth Shakman Hurd observe that ‘religious freedom, not unlike other fundamental freedoms invented in the last century, is a contested and multivalent historical construct that has taken on new lives of its own’.^33^ Their research project explored the historical and global diversity of religious freedom, with particular emphasis on political implications. Relatedly, Hurd’s book, *Beyond Religious Freedom: The New Global Politics of Religion*, demonstrates that Western promotion and dissemination of ‘religious freedom’ has often disempowered minority groups and fomented sectarian strife.^34^ In an effort to historicise recent scholarship, Samuel Moyn emphasises that religious freedom began as a Christian tool against communist secularism and only recently became a secular tool against minorities.^35^ Responding to much of the critical secularism scholarship on religious freedom, Philpott and Shah argue that

the new critics’ claim that religious freedom is tightly bound to Protestant, privatised religion and the secular state—and is thus an exclusively Western idea that can only be imposed on the rest of the world—is riddled with difficulties.^36^

Contrary to this assertion, we demonstrate that a dominant, contemporary understanding of religious freedom does restrict Jewish and Muslim practices—at least in Western states, and possibly in non-Western states as well. States apply religious freedom in varying ways because the underlying notion of religion (as religiosity, religious law and religious group) is variable and has different implications for distinct traditions.

While each individual secular state has a specific history, political situation and other peculiarities that shape religious freedom, these particularities do not alter the discriminatory potential of ‘religious freedom’, as recent scholarship has demonstrated. While most studies have focused on private aspects of religion, we shift attention towards the public sphere and the state’s role in shaping religion. We contribute to existing scholarship (within and beyond critical secularism studies) that has established that ‘religious freedom’ is used to discriminate against minorities. In a recent report on freedom of religion, the International Development Law Organization observes that ‘the law has far too often been used to restrict freedom of religion or belief and, in particular, its exercise by members of minority groups’.^37^ For instance, recent decisions by the ECtHR reflect a Christian bias by permitting Christian symbols and limiting Muslim ones.^38^ We concur with these observations; indeed, we have written specifically about how secular states generate Judaeophobia and Islamophobia by depicting Jews and Muslims as threats to public order.^39^ Even so, we acknowledge that many scholars have challenged the growing body of critical secularism scholarship in the area of religious freedom for failing to recognise its positive implications. By way of example, some scholars contend that religious freedom is not a tool used by the majority to dominate minority groups, but rather—like other human rights—a protective tool for minority groups. Philpott and Shah, for instance, claim, ‘religious freedom has far more often been a weapon of the weak than a technology of the strong’.^40^ We cannot resolve the empirical question of whether religious freedom has been, in general, more detrimental or advantageous to minorities.

While critical secularism studies has established the relationship between Christianity and the modern legal doctrine of religious freedom, it has done so without fully engaging non-Christian traditions. Our case study on circumcision in Germany reveals *how* religious freedom is used to restrict minorities by elaborating the differences between post-Christian secular legal reasoning, on the one hand, and Jewish and Islamic legal traditions, on the other. States secularise traditions by defining them as ‘religions’ and introducing them into legal discourse and state regulation through three dimensions that are simultaneously related and conflicting. Secular law frames religion within a ‘problem-space’. Hussein Agrama explains that ‘what best characterises secularism is not a separation between religion and politics, but an ongoing, deepening, entanglement in the *question* of religion and politics’.^41^ Secular states construct a problem-space in which the definition of religion, although multifaceted and fluctuating, is framed by a triangle of religiosity, religious law and religious group.^42^ Many scholars have explored how law constructs religion as individual belief. We propose that the implications of understanding religion as individual belief differ for Muslims and Jews, as compared to Christians. Although secular law elevates a definition of religion as individual belief, it also recognises religion as practice, but primarily when that practice is evident in positive law.^43^ Likewise, secular law privileges a definition of religion as individual, but also recognises religion as communal (or collective) when it marks particular groups as a public threat. The secularisation triangle becomes evident when we focus on Jewish and Islamic traditions, rather than normative Christian perspectives. In the next section, we explore the overlaps and implications of the secularisation triangle for our case study.

## 3. A Case Study on Germany’s Recent Circumcision Controversy

On 26 June 2012, a German district court in Cologne (*Landgericht Köln*) ruled that circumcising young boys causes grievous bodily harm.^44^ The case centred on a four-year-old Muslim boy who experienced medical complications after his parents had him circumcised by a doctor.^45^ Two days after the procedure, the incision started bleeding and the mother and son went to the emergency room. Based on the complaint of the attending ER physician, the public prosecutor charged the doctor who performed the circumcision with battery. While the trial court (Amtsgericht Köln) did not find the physician guilty, on appeal, the Cologne district court (Landsgericht Köln) concluded that circumcision of infants is battery with a dangerous instrument.^46^ The district court declared that ‘the right of the parents to raise their child in their religious faith does not take precedence over the right of the child to bodily integrity and self-determination’.^47^ This decision left Muslim and Jewish parents under suspicion of causing bodily harm to their sons.

The district court’s ruling provoked strong objections across political party lines and in the international arena.^48^ Muslim and Jewish groups protested the decision vehemently.^49^ Chancellor Angela Merkel famously declared ‘I do not want Germany to be the only country in the world where Jews cannot practise their rituals, otherwise we will become a laughing stock’.^50^ Appealing the decision was not possible because the court had acquitted the physician for ignorance of the law. It was clear that the legislature would have to intervene, but it was unclear how. Within six months, the German parliament (Deutscher Bundestag) passed a law that allowed circumcision for non-medical purposes; it permitted non-medical experts to perform circumcision up to the age of six months, but required medical professionals to perform the procedure after that age.^51^ Although the legislation was framed in general terms, it created, *de facto*, a significant disparity between Jews and Muslims: Jews could continue to practise their tradition (of circumcision on the eighth day by a community circumciser), whereas Muslims (who in Germany commonly circumcise at a later age) would be required to rely on a medical professional. While opponents of circumcision claim that it is not medically necessary, the court did not address medical debates about circumcision. As is well known, there is no medical consensus and some medical professionals advocate for circumcision as medically preferable.^52^ Indeed, there are multiple medical arguments concerning circumcision and the choice of one over the other is largely political, rather than purely scientific.

Although medical necessity was not addressed, the German legislature recognised ‘religious, cultural, or prophylactic reasons’ for circumcision.^53^ Notably, in a December 2012 survey, a German polling organisation, Infratest, found that 70% of Germans opposed the Bill’s authorisation of male circumcision.^54^ While this poll, as all polls, should be viewed with scepticism, it does suggest that the matter of male circumcision in Germany is not resolved and that the state, the majority population and minority groups will continue to debate its legality.^55^ Indeed, a research division of the German legislature recently discussed potential gender inequality in the law’s facilitation of male circumcision while banning female circumcision.^56^ These debates, even as they generate coalitions between Jews and Muslims, take place within the German state’s secularisation triangle.

### A. *Religiosity: Private Belief, Individual Right and Autonomous Choice*

On its face, the German controversy concerns the tension between the bodily integrity of the child and the religious freedom—or, rather, the religious liberty—of parents to raise their child as a Muslim or Jew. Yet, the Court interpreted religious freedom as being *against* circumcision. The district court argued, inter alia, that a child should be allowed to choose circumcision, asserting that ‘circumcision changes the child’s body permanently and irreparably. This change runs contrary to the interests of the child in deciding his religious affiliation independently later in life.’^57^ The court assumed that circumcision would prevent an individual from exercising religious freedom—a surprising presumption for which the court provided no supporting evidence.^58^ In other words, the court did not explain why being circumcised would prevent someone from changing his religion.^59^ In addition, the court declared: ‘Consent by the four-year-old was not given and could not be given due to a lack of intellectual maturity. The consent of the parents was given, but could not justify the infliction of bodily harm.’^60^ The court did not address why a child’s consent is unnecessary for comparable or routine medical procedures (such as vaccines) that also affect the body.^61^ Similarly, the court did not explore why a child’s consent is unnecessary for gender modification of intersex babies.^62^ The German court emphasised consent because it views religiosity as a matter of private belief, individual right and autonomous choice.^63^

The secular (Christian-mediated) understanding of religiosity is based on a notion of autonomous choice. The notion of religiosity as choice diverges from how Jewish and Islamic traditions historically constructed the practice of circumcision. For both Jews and Muslims, one is born into a tradition in which male circumcision is a normative practice; one does not choose to become Jewish or Muslim and then choose to manifest this choice by being circumcised. Indeed, with the exception of apostasy, even extreme violations of law do not release one from the community. The Babylonian Talmud famously declares, ‘A Jew, even though he has sinned, is a Jew’.^64^ Historically, exiting from Jewish or Muslim communities was possible only through a public declaration rejecting the community. The court’s construction of religiosity as a matter of individual belief, one that lies in the inner conscience of the individual, appears to echo Christian discussions of circumcision. In Galatians, Paul asserted,

^2^Listen! I, Paul, am telling you that if you let yourselves be circumcised, Christ will be of no benefit to you. ^3^Once again I testify to every man who lets himself be circumcised that he is obliged to obey the entire law. ^4^You who want to be justified by the law have cut yourselves off from Christ; you have fallen away from grace. ^5^For through the Spirit, by faith, we eagerly wait for the hope of righteousness. ^6^For in Christ Jesus neither circumcision nor uncircumcision counts for anything; the only thing that counts is faith working through love.^65^

Similarly, the German court emphasised the child’s belief and autonomous choice over law or ritual practice. In contrast, Jews and Muslims conceptualise tradition as belonging to a community.

Presumably, for the German Court, baptism did not raise a similar concern to that of circumcision because it does not leave a mark on the body.^66^ Not coincidentally, Paul advocated that the spiritual circumcision underlying baptism superseded circumcision specifically and Jewish legal commandments more generally.^67^ Connecting historical Christian ideas with contemporary issues, William Galston suggests that ‘the human rights community’s insistence on adult consent as a necessary source of authorisation represents a secularised version of the Protestant rejection of infant baptism’; accordingly, he questions if ‘it is an accident that the epicenter of antipathy to infant circumcision is located in heavily Protestant northern Europe’.^68^ Galston implies that a long history of Christian debates about circumcision shapes contemporary secular debates. The characterisation of Judaism as an overly legalised tradition was a significant component of early Christian self-articulation and polemics against Jews during antiquity and beyond.^69^ Yet, even while overruling Jewish commandments, early Christian figures acknowledged those commandments as a legitimate source of law. The modern state assumes both aspects of early Christian polemics: elevating belief over law and recognising biblical law.

By adopting a notion of religiosity based on Christian traditions, the German court also adopted perspectives on the human body that are not neutral, but rather emerge from those same traditions. The German court explicitly stated that ‘Circumcision for the purpose of religious upbringing constitutes a violation of physical integrity, and if it [circumcision] is actually necessary, it is at all events unreasonable’.^70^ The German court’s notions of bodily harm, physical integrity and unreasonableness may be a transformation of Pauline Christian arguments against circumcision. In both the Jewish and Islamic traditions, circumcision is not bodily harm, but rather bodily improvement. The rabbinic Jewish tradition views circumcision as perfecting the body.^71^ Similarly, orthodox Muslims articulated an understanding of circumcision as part of ‘the natural predisposition’ or ‘beginning state’ (*fiṭrah*) of the body.^72^ Thus, orthodox Muslims understand circumcision as returning the body to its most natural state. In both the Jewish and Islamic perspectives, the notion of what is natural and unnatural is the reverse of prevalent secular assumptions. Describing circumcision as ‘bodily harm’ reflects Christian bias.

A state’s definition of religiosity as private belief synthesises specifically antinomian ideas from the Christian tradition and novel aims of the modern state. Robert Yelle explains that Christian antinomianism, which precedes Protestantism,

is the idea that religion is a matter of the spirit and not of law or ritual. This view of religion, which was originally applied by Saint Paul to distinguish Christian ‘grace’ from Jewish ‘law’ (Rom. 6:14), was extended by Protestant theologians, who further valorised religious belief over ritual practice.^73^

It is widely recognised that states often construct religion as individual belief. For instance, Benjamin Berger illustrated that secular law constructs religion as private, individual and autonomous.^74^ We contend that religiosity, one angle of the secularisation triangle, should be expounded as private belief, individual right and autonomous choice.

First, secular law defines religiosity as private belief.^75^ For instance, the ECtHR differentiates between ‘holding’ a religious belief and ‘manifesting’ that belief, declaring that secular law can only limit the latter.^76^ Similarly, Sullivan has demonstrated that US judges perceive religion as a matter of private ‘views’, rather than acts.^77^ The US Supreme Court has not explicitly defined religion, but it has repeatedly indicated that ‘sincerely held beliefs’ are markers of religion.^78^ Talal Asad explains that constructing religion as belief ‘is a modern, privatized Christian [perspective] because and to the extent that it emphasizes the priority of belief as a state of mind rather than as constituting activity in the world’.^79^ Secular law’s differentiation between belief and act reflects Protestant Christian ideas and the desire of the modern state to police the public sphere by confining minority communities to the private sphere.

Secondly, secular law identifies religiosity as an individual right.^80^ Although secular law recognises religious groups, it commonly treats them as groups of private individuals, rather than as public groups.^81^ Put differently, secular law marks religion as non-public and, by extension, non-political. For instance, article 9 of the ECHR limits freedom of religion ‘in the interests of public safety, for the protection of public order’. The ECtHR case of *Valsamis v Greece* illustrates how secular law regulates the border between the individual and the public.^82^ The ECtHR found that Greece did not violate the applicant’s article 9 rights by punishing a student for refusing to participate in Greece’s National Day because it celebrates warfare, which the applicant argued is a violation of her religious beliefs.^83^ In a guide to article 9, the ECtHR’s Research Division declared that the nationalist parade did not ‘offend’ the applicant’s beliefs, but rather fulfilled the public interest and even, inexplicably, her religious objectives.^84^ It is striking that this case so explicitly identifies the public, political ritual of the modern nation-state as negating an individual right to freedom of religion. This case, and others, indicates that secular law constructs the individual right of freedom of religion as a non-political right.

Thirdly, as many scholars have observed, secular law defines religiosity as a matter of autonomous choice, although most followers of Jewish and Islamic traditions do not experience it as such.^85^ The assumption of choice is acutely evident in cases concerning the display of religious symbols. The ECtHR views the display of ‘religious symbols’ as ‘bearing witness’ and ‘a manifestation of … religious belief’.^86^ In other words, secular law interprets religious symbols as belief-based choices. In the cases *Dogru v France* and *Kervanci v France*, the ECtHR accepted the French government’s claim that the students ‘refused’ to remove their headscarves, which assumes that wearing the headscarf is a choice.^87^ In these and other cases, the ECtHR construes symbols or clothing as equivalent to optional accessories. Rather than a premeditated choice between equally available alternatives, many women wear headscarves because of customary traditions they consider to be fixed and unalterable. Indeed, many women perceive the wearing of a headscarf as an obligation required by law. Secular law uses the notion of choice to depict traditional practices as discretionary.

The secular state’s definition of religiosity as private belief, individual right and autonomous choice conflicts with Jewish and Islamic traditions. Contrary to secular ideas, Jewish and Islamic traditions entail public acts (ie taking place in the public sphere) and collective obligations. Both Jewish law and Islamic law recognise a category of obligations that fall on the entire community, rather than on individuals. Still, a state’s definition of religiosity as private belief, individual right and autonomous choice has disparate implications for Jews and Muslims. In contemporary Western states, Muslims who do not conform to the state-sanctioned understanding of religiosity are more likely to be considered threats to the public sphere. For instance, Muslim women’s wearing of headscarves is a source of social and legal anxiety precisely because it is misconstrued as a public act, social imposition and non-autonomous choice.^88^ Yet, Muslim women’s wearing of headscarves is incongruent with the notion of religiosity. The same is true of Jewish and Muslim circumcision.

### B. *Religious Law: A Divinely Ordained Legal Code*

Although state law primarily defines religion as religiosity, it also constructs a notion of religious law that is based on a written legal code (ie positive law). The German court declared that ‘the parental right of education is not unacceptably diminished by requiring them to wait until their son is able to make the decision himself whether to have a circumcision as a visible sign of his affiliation to Islam’.^89^ The German court based this claim on the fact that there is no fixed Islamic date for circumcising a child. Islamic legal sources provide multiple, authoritative opinions on the timing of circumcision, ranging between seven days and the onset of puberty.^90^ This range of opinions on the timing of circumcision results from there being no Qurʾānic verse that explicitly mentions or requires circumcision. While Islamic scripture does not proscribe a specific age for circumcision, the orthodox Islamic legal view is that puberty constitutes the upper age limit for circumcision.^91^ In Germany, Muslim parents practise circumcision between the child’s infancy and the age of puberty (approximately 13 years), in accordance with orthodox Islamic law. Consequently, the German court erred in assuming that waiting until the son ‘comes of age’ would not violate Islamic law.^92^ Although the German court did not specify ‘coming of age’, it would probably be 14 years of age, which German law recognises as the age of religious independence.^93^ German law professor and anti-circumcision activist Holm Putzke advocated that the age of consent for circumcision should be 16 years.^94^ (By way of comparison, German law prohibits ear piercing or tattooing below the age of 18 years, unless there is parental authorisation between the ages of 16 and 18 years.^95^) Since the German court’s definition of ‘coming of age’ is post-puberty, it violates orthodox Islamic law. Because the state primarily recognises a written and verifiable legal code as binding, the court ignored the multivocality of Islamic law. Thus, the court fused Christian ideas with its objective of legal certainty when it presumed that only codified, written law validates practices.

In addition to the German court, the German legislature has considered the timing of circumcision. The German Civil Code specifies:

In the first six months after the child is born, circumcision may also be performed pursuant to subsection (1) by persons designated by a religious group to perform this procedure if these persons are specially trained to do so and, without being a physician, are comparably qualified to perform circumcisions.^96^

The German Legislature and Ethics Council formally justifies the distinction of six months based on the lower sensation of pain and the lower medical risks for circumcision at an early age.^97^ However, six months is an arbitrary age to create a threshold for pain and there is no medical research to support it. The German legislation appears to cater to Jewish practices of fixed circumcision timing, thereby giving less weight to Muslim practices. German legal actors probably perceive Jewish timing of circumcision as legal and binding because it occurs on a clearly stipulated day, in conformity with Leviticus 12:3 (‘On the eighth day, the flesh of his foreskin shall be circumcised’).^98^ In addition, local and international pressures, as well as potential accusations of anti-Semitism, probably influenced the legislation. When states evaluate Islamic law, they often do so by comparing it to Jewish law. In the case of circumcision, this comparison contributes to viewing Islamic circumcision practices as customary, rather than binding, and consequently as non-legal. Accordingly, the Jewish timing of circumcision was taken as uncontested and unquestionable, while Muslim traditions did not receive similar respect.

State law both constructs and enforces a distinction between binding religious laws (that the state affords legal protections) and customs (that the state denies legal protections). This is why some state courts have demanded that minorities prove that a given practice is not only a custom, but also a legally inscribed duty. For example, some (secular) courts have declared that animal slaughter on Muslim holidays is merely a custom and not an obligation.^99^ Similarly, some legal commentators have argued that the wearing of face veils is merely a custom and not obligatory.^100^ Recently, a state actor in the UK differentiated between the obligation to wear a headscarf after puberty and the custom of wearing one before puberty; a headteacher at a school in East London prohibited girls under the age of eight from wearing headscarves, claiming that orthodox Islamic law only requires headscarves for girls after puberty.^101^ When secular law requires evidence of positive law in order to uphold religious freedom, it informs a notion of religious law that limits traditions by excluding plurality and heterodoxy.

Secular law’s demand for positive law, in the form of a divinely ordained legal code, is far from neutral. The secular state’s imposition of religious law results from the synthesis of certain Christian ideas and the state’s interest in legal certainty. Some early Christian writings characterised Judaism as overly legalistic, even while acknowledging the divine basis of Jewish law.^102^ Just as in late antiquity Christianity differentiated between biblical law and rabbinic law, the state differentiates between positive law and custom.^103^ Analogously, the state discriminates against traditions whose oral law, local diversity, customs and heterodox practices do not fit within the state’s demand for a legal code. In the early modern era, some Christian thinkers marked a clear distinction between ‘true religions’ grounded in belief and ‘imperfect religions’ grounded in practices, rituals and legal codes.^104^ Drawing upon specifically Protestant ideas, Kant formulated a view that continues to influence contemporary disputes.^105^ According to Kant, (Protestant) Christianity is the paradigm of a perfected religion, whereas Judaism (and, by extension, Islam) are imperfect religions.^106^ Kant elaborated a distinction between *Vernunftreligion* (a true religion based on reason) and *Kirchenreligion* (a church religion based on religious law (*Glaubenssätze*) and ritual).^107^ Similarly, states legally protect imperfect religions when their religious laws stem from a clear authority and have unambiguous or undisputable content. Yet, Jewish and Islamic legal traditions are not positivist; mandated law is only one component of both legal traditions.

Admittedly, the distinction between custom and mandated law is meaningful even within Jewish and Islamic traditions, since both recognise custom as a source of law.^108^ Nevertheless, secular states and traditions define the distinction between custom and mandated law differently. First, the distinction between custom and mandated law does not correspond to the distinction between written and unwritten law; customs may be written while legal obligations may be oral. What makes certain norms more obligatory than others is not a procedural standard, but rather a dynamic process within traditions.^109^ Secondly, within Judaism and Islam, customs are often obligatory and at times as obligatory as mandated laws. Custom is a normative category and is never ‘mere’ custom. Thirdly, secular states apply the distinction between custom and obligation inconsistently, or in ways that are prejudiced against minorities. For instance, some courts have used the claim that a minority practice is ‘mere custom’ in order to grant legal protections to majority practice. By way of example, the ECtHR approved the hanging of a cross in Italian schools based on the reasoning that if the cross is ‘mere custom’, then it is of cultural rather than religious significance, and its display in public spaces does not violate the separation of state from religion.^110^ The ECtHR’s decision in this case contrasts with other cases in which a minority practice has been prohibited because it was ‘mere custom’. Moreover, this is not an isolated case of a Christian practice being depicted as a custom. For instance, the government of Bavaria—a predominantly Catholic region in Southern Germany—ordered the hanging of crosses in the entrances of government buildings as a reflection of the region’s cultural customs.^111^ Of course, states do sometimes recognise customary law that is not codified; the point is that the state selectively requires evidence of religious law from minority groups.

Secularism’s definition of religious law has disparate implications for Jews and Muslims. By virtue of a variety of historical reasons, the Islamic legal tradition is significantly more pluralistic than the Jewish legal tradition.^112^ Correspondingly, there is more multivocality and variation within orthodox Islamic law than orthodox Jewish law. As a result, when the state demands evidence of religious law (as it does in some religious freedom cases), it discriminates against Muslims more than it does against Jews. Indeed, Jews, especially in the tight-knit communities of central and eastern Europe, developed a narrower legal orthodoxy than Muslims. Moreover, the secular state’s demand for religious law should be placed within a broader history of colonial codifications of Islamic law as mechanisms of colonial control.^113^ Ironically, recent Islamophobic legislation against ‘sharia law’ (sic) incorrectly presumes that Islamic law is a positive legal code and identifies its followers as a public threat.^114^

### C. *Religious Groups: Public Threat*

As the previous sections have illustrated, the German court has simultaneously construed circumcision as a matter of religiosity (private belief, individual right and autonomous choice) and religious law (a practice by a divinely ordained legal code). In addition, we propose that legal and public opposition to circumcision is related to stereotypes of ‘religious violence’, generally, and to a perceived threat of Muslims, specifically.^115^ Many opponents of circumcision portray it as a violent act against a defenceless child at the mercy of his parents and religious community. In Europe, circumcision (and, relatedly, animal slaughter) is sometimes associated with barbarism. For instance, some scholars specialising in legal and medical ethics claim that circumcision ‘began as a sacrificial religious ritual and painful rite of passage’.^116^ In addition, some opponents of male circumcision liken the practice to female circumcision in an attempt to delegitimise both practices. Some German media portrayed the court’s prohibition on male circumcision as a natural continuation of the state’s pre-existing prohibition on female circumcision.^117^ The comparison between male and female circumcision is important because it demonstrates how the state’s classification of ‘Muslim’ can signal a threat to public order.^118^ Although a majority of Muslims do not practise female circumcision, Islamophobic commentators depict it as a manifestation of ‘Muslim violence’. In the specific case brought before the German court in Cologne, some media focused attention on the identity of the individuals involved: the child’s North African mother spoke little German and the physician immigrated from Syria, although he was trained in Germany.^119^ The recent German controversy on male circumcision elucidates how the prejudicial depiction of Muslims as public threats influences legal and political debates.

While the German court did not raise public threat as a consideration, it likely explains why this case entered the German legal system. The German court (and later the German parliament) may have differentiated between Jewish circumcision and Muslim circumcision because of the state’s classification of Muslims as a threat to public order. The case that was brought to the German court concerned a Muslim doctor’s circumcision of a Muslim boy at the behest of his Muslim parents.^120^ If the case were of a Jewish doctor’s circumcision of a Jewish boy at the behest of his Jewish parents, the court would likely have decided the case differently. It is probable that the history of the Nazi genocide of Jews motivated the German parliament to pass legislation permitting circumcision. We suggest that the legislative distinction made between circumcisions during and after the first six months of a boy’s life is not a medical or ethical matter, but rather a limitation on the minority group (Muslims) that the state views as a threat to public order.^121^

The state’s classification of Jews and Muslims as religious groups obfuscates the reality that being Jewish and being Muslim are not equivalent categories.^122^ The secular conversion of the Jewish and Islamic traditions in the form of ‘religions’ has obscured their historical expressions. Indeed, the specific practice of circumcision illuminates why ‘Jewish’ and ‘Muslim’ are not analogous categories.^123^ Rabbinic traditions on circumcision interpret the blood of circumcision as marking the entrance of a Jew into the covenant between God and the Israelites.^124^ In contradistinction, Islamic traditions interpret circumcision as a practice that is necessary for bodily cleanliness. Consequently, the role of circumcision in Jewish versus Muslim communal belonging is not equivalent. This is particularly evident in the conversion process. Circumcision was so central to Jewish conversion that rabbis debated the necessity of a ‘symbolic circumcision’ for converts who were already circumcised.^125^ There is no comparable discussion of symbolic circumcision in Islamic legal literature because the orthodox view does not require converts to be circumcised. Jewish and Islamic traditions about circumcision indicate not only dissimilar practices, but also disparate understandings of belonging. The state classification of Jews and Muslims as a public threat has normalised the false assumption that they are corresponding groups.

States often associate minority group practices as a threat to public order; women’s headscarves are an important example. Analysing the ECtHR’s decision concerning women’s headscarves, Nehal Bhuta demonstrates that the core issue is the state’s depiction of veils as threats to public order and as ‘harbingers of sectarian strife which undermine democracy’.^126^ He argues that the ECtHR engages two versions of religious freedom, observing that ‘the first concept focuses on religion as a question of individual belief, while the second concept is concerned with religion as a sectarian association that may undermine social cohesion’.^127^ These two versions of religious freedom explain the ECtHR’s legal reasoning, since, as Bhuta explains, ‘to wear a religious symbol which might be understood as an expression of political Islam is to threaten secularism and equality, justifying a complete prohibition of the headscarf in public institutions such as universities’.^128^ Many states classify headscarves as a threatening minority practice. This presumed threat to public order is sometimes articulated as the ‘freedom from religion’ or the rights of others not to be exposed to religion in the public sphere. Admittedly, states often classify groups with relatively benign goals, including gathering statistical and demographic information; nevertheless, state bureaucracies have used religious affiliation—as well as other state classifications, such as gender, ethnicity and race—as an indicator of public threat.

When states associate Jews (primarily in the past) and Muslims (at present) with violence, they contribute to the myth that religion is a primary or unique cause of violence.^129^ William Cavanaugh explains:

The myth of religious violence helps to construct and marginalise a religious Other, prone to fanaticism, to contrast with the rational, peace-making, secular subject. This myth can be and is used in domestic politics to legitimate the marginalisation of certain types of practices and groups labeled religious.^130^

Nonetheless, there are notable distinctions between how Jews and Muslims are depicted as public threats: Jews are viewed as conspiratorial actors, while Muslims are viewed as violent actors.^131^ Jewish violence in the 19th century was depicted as sinister and as taking place behind doors; the archetypical Judeophobic accusation is the blood libel. In contrast, Muslim violence is often portrayed as public; the archetypical Islamophobic accusation is terrorism.^132^ An illuminating case comes from the European context of animal slaughter.^133^ Europeans associated Jewish slaughter with the blood libel, whereas they associated Muslim slaughter with terror attacks. The most common accusation levelled against Jewish and Muslim slaughter concerned its inhumane character. Judaeophobes and Islamophobes associate ritual slaughter with Jewish and Muslim violence, implying that adherers of the practice are prone to even more extreme violent acts.

Secular states stigmatise Jews and Muslims as being incompatible with (secular) public values and as being threats to political authority and public security.^134^ (The stigmatising of Muslims and Jews is the first step towards what many scholars describe as their racialisation or ethnicisation.^135^) The International Development Law Organization has observed that

Recent and historical experience has amply demonstrated that restrictions on religious expression, often defended by the State on grounds relating to national security, public order, or even human rights, could in fact be intended to target and marginalize particular minorities on a discriminatory basis.^136^

Secularism’s construction of religious groups restricts a dimension of Jewish and Muslim identities that does not fit within the secular definition of religion and therefore challenges state hegemony: the political. In the pre-secular world, Jewish and Muslim identities were inevitably political.^137^ By contrast, in a modern secular state, citizenship is presumed to transcend all other collective identities; thus, Jewish and Muslim collective identities are conceived as posing a threat to secular state security.

The modern state tolerates its religious subject so long as the private beliefs of autonomous individuals do not threaten political authority. In contradistinction, the modern state views public acts of collectivities as a threat to political authority. This is evident in a case concerning a Hasidic Jewish community in Montreal. When the community sought to maintain an *eruv* (a rabbinically required symbolic line delineating Jewish space for Shabbat observance), they were confronted with secular opposition to their presumed invasion of public space.^138^ The installation of an *eruv* is a public act that demonstrates communal membership and submission to rabbinic law. Therefore, the *eruv* challenges the secular notion of religiosity and religious group. Not surprisingly, the *eruv* has been the subject of controversy in multiple secular states.^139^

Secular intolerance for those who do not fit within its legal definition of religion can transform into fear and repression. When secular law exerts its authority over minorities, it frequently identifies them as public threats. Count Clermont-Tonnerre, a French aristocrat involved in the French Revolution, was well aware of the threat of collectivities when he stated: ‘We must refuse everything to the Jews as a nation and accord everything to Jews as individuals.’^140^ His use of the term ‘nation’ is instructive: a nation is a political collectivity. It is no coincidence that many Islamophobes in the United States claim that Islam is a political movement rather than a religion, and therefore should not be protected under the First Amendment.^141^ Similarly, debates in Denmark characterise Islam as a ‘law religion’, suggesting that Muslim adherence to Islamic law supersedes Danish law.^142^ From a secular perspective, the potential for Jews or Muslims to be political is a public threat.

Secular states often characterise minorities as violent threats because the state is focused on maintaining its monopoly on the use of violence. In the contemporary context, the notion that religious groups pose a public threat has a disparate impact on Jews and Muslims. The state’s classification of Jews curtails their religious freedom; by comparison, the state’s classification of Muslims limits their citizenship. James Renton and Ben Gidley observed that

the global infrastructure of surveillance, incarceration and killing that is today focused on Muslims has no precise precedent. The closest we come to it in history, in essence though not in scale, is the structure of the anti-Jewish surveillance and control apparatus of the Nazi state.^143^

Regardless of the accuracy of this analogy, it is undeniable that the specific category ‘Muslim’ has been used for risk assessment and profiling, with significant implications for Muslim minorities.^144^ The secular state uses religious group identity, grounded in Judaeophobic and Islamophobic images of violence and danger, as a classificatory mechanism.^145^

## 4. A Historical Perspective on State Secularisation of Circumcision

Long before German courts scrutinized Muslim circumcision, Jewish circumcision provoked legal, religious and medical debates in Germany.^146^ An early dispute erupted in the 1840s when a handful of Jewish fathers refused to circumcise their sons.^147^ The parents, some of whom were medical physicians, were concerned with reported cases of death following circumcision. The risk to public health along with a decline in observance led Jewish parents to refrain from circumcising their sons. Nonetheless, they demanded that their sons be registered as members of the Jewish community. Though Judaism is traditionally matrilineal, many of the rabbis involved refused to register uncircumcised boys based on maternal heredity, claiming that circumcision was a necessary condition for being a member of the Jewish community. (Rabbinic law recognises an uncircumcised Jewish man (*ʿarel*) as Jewish, but ritually impure; thus, despite its significance, circumcision was not perceived as obligatory to Jewishness in the premodern world.^148^) The debates that ensued within the German Jewish community soon found their way to German public authorities. Concerned fathers turned to the state’s non-Jewish authorities, pleading with them to order the rabbis to register their sons.

Some German authorities respected the decisions of the Jewish community, while others instructed the community to register the non-circumcised boys as Jews. An interesting chain of events took place in the small town of Hürben in southern Germany in 1845. Local parents who refused to circumcise their sons demanded that the Orthodox rabbi, Joachim Schwarz, register the boys as Jews; they turned to the state authority to force the reluctant rabbi to comply.^149^ Rabbi Schwarz justified excluding the uncircumcised boys from the community by claiming that circumcision was akin to baptism and therefore a precondition for entering the Jewish community. By the 1870s, the controversy surrounding circumcision had subsided. The ostensible public health issues were resolved in a compromise that appeased even the Orthodox-leaning rabbis. Henceforward, membership in the Jewish community was determined on confessional grounds, so that the Orthodox congregations, which wished to do so, could split from the central organisation and regulate membership in the community according to their own understanding, as the Jewish community in Frankfurt in fact did. These changes in the configuration of the German Jewish community constitute what Leora Batnizky has described as the transformation of Judaism into a religion that resembles Christianity.^150^ We propose that an analogous process is occurring now as states secularise the Islamic tradition and transform it into a religion.

In response to the state’s definition of religion as religiosity, religious law and religious group, Muslims—like their Jewish predecessors—in Germany defended circumcision in the state’s terms. For example, a physician and Muslim community leader in Germany publicly argued against German popular assumptions that Muslim circumcision is non-compulsory.^151^ In doing so, he proposed that Muslim circumcision is obligatory and divinely ordained, mimicking the state’s expectations and disregarding aspects of the Islamic tradition that are contrary. If we understand German Muslim responses to the circumcision controversy as part of a secularisation process, then one consequence of this process is erasing the reality that Muslim circumcision was historically not a universally obligatory practice.^152^ The debate about Muslim circumcision in Germany is an indication of an ongoing process of secularising the Islamic tradition by converting it into a religion that the state can classify and control.

## 5. *Conclusion*

Germany, like other modern liberal states, is not religiously neutral; rather, the state is shaped by its specific history and its interactions with both majority and minority groups. Germany’s Christian heritage and Nazi past informs its distinct relationships to Jews, on the one hand, and Muslims, on the other. Circumcision is a particularly effective case study because it reveals how secular frameworks are rooted in historical narratives about modern notions of religion as religiosity, religious law and religious group. Focusing on the trilateral relationship between the post-Christian state and its Jewish and Muslim minorities is illuminating. Underlying contemporary debates is a story about circumcision as a Jewish ritual that Christians abrogated and Muslims adopted. In this modern, linear construction, Jewish circumcision is the precedent and standard for Muslim circumcision. However, this simplified framing misconstrues the horizontality of historical circumcision practices and the haphazardness of circumcision in the Islamic tradition. Whereas premodern Jews and Muslims articulated their identities in varying ways through circumcision, modern Jews and Muslims are being disciplined through secular limitations on circumcision. Modern secular law delineates the boundaries of Muslim circumcision by comparing it to Jewish circumcision. In a predominantly Christian society, Jewish circumcision becomes the standard to which Muslims must adapt.

Conventional ways of understanding the modern state’s treatment of religion are overly limiting: neither the liberal view that the modern state should permit religious freedom nor the critical view that the state should not discriminate against religiously observant citizens accounts for the more fundamental issues that we have explored in this article. The secularisation triangle and the trilateral relationship between the state and its Jewish and Muslim minorities elucidate how ‘religious freedom’ can operate as a mechanism of state control over minorities. Historical investigations reveal that Jewish and Islamic traditions cannot be bound within the limiting notions of religiosity, religious law and religious group. Jewish and Islamic traditions do not fit into the secularisation triangle. States secularise traditions. In doing so, states shape how minorities both conceptualise and practise their own traditions. We invite scholars of law and religion to test the general patterns we have identified in this article to their areas of specialisation.

## Footnotes

1 On secularism and secularisation, see José Casanova, *Public Religions in the Modern World* (University of Chicago Press 1994); Jürgen Habermas and Joseph Ratzinger, *The Dialectics of Secularization: On Reason and Religion* (Brian McNeil tr, Ignatius Press 2007); Charles Taylor, *A Secular Age* (Harvard UP 2007).

2 See classifications of types of secular states in Ahmet T Kuru, ‘Passive and Assertive Secularism: Historical Conditions, Ideological Struggles, and State Policies toward Religion’ (2007) 59 World Politics 568, 570; Peter O’Brien, *The Muslim Question in Europe: Political Controversies and Public Philosophies* (Temple UP 2016) 144.

3 On the immeasurability of secular states, see Hussein A Agrama, ‘Sovereign Power and Secular Indeterminacy: Is Egypt a Secular or a Religious State?’ in Mateo Taussig-Rubbo, Robert A Yelle and Winnifred Fallers Sullivan (eds), *After Secular Law* (Stanford Law Books 2011).

4 Mahmood and Danchin explain ‘This normative structure of the right to religious liberty explains the close intertwining of the religious and the secular, whether in the Middle East or Europe, and illustrates the error in viewing these as separate or opposing worldviews’: Saba Mahmood and Peter G Danchin, ‘Immunity or Regulation? Antinomies of Religious Freedom’ (2014) 113 South Atlantic Quarterly 129, 155. See also Zachary R Calo, ‘Constructing the Secular: Law and Religion Jurisprudence in Europe and the United States’ (2014) EUI Working Paper RSCAS 2014/94.

5 We do not claim that modern nation-states are the only form of states. Bevir and Rhodes explain that ‘The state, or pattern of rule, is the contingent product of diverse actions and political struggles informed by the beliefs of agents rooted in traditions’: Mark Bevir and R.A.W. Rhodes, *The State as Cultural Practice* (OUP 2010) 99.

6 Notably, international legal institutions, such as the European Court of Human Rights, share the legal reasoning of secular states.

7 We differentiate between tradition and religion based on our extensive previous scholarship about premodern Jewish and Islamic traditions. We base our definition of tradition primarily on Mark Bevir, ‘On Tradition’ (2000) 13 Humanitas 28, 38. See also Yaacov Yadgar, ‘Tradition’ (2013) 36 Human Studies 451.

8 Notably, we do not intervene in the complex issues of who represents a tradition, who should determine belonging in a minority community or who should protect minorities within minority traditions. Because this article concentrates on Christian-majority states in the West, ‘minority’ refers to Jews and Muslims. However, some of the dynamics we delineate are observable in both Jewish majority and Muslim majority jurisdictions.

9 See eg *Employment Division, Department of Human Resources of the State of Oregon, et al v Alfred Smith* 494 US 872, 110 S Ct 1595 (holding that the government has a compelling interest in prohibiting the religious use of peyote; ‘neutral law’ does not violate the Free Exercise clause of the First Amendment).

10 On the similarities between European and US religious freedom jurisprudence, see Zachary R Calo, ‘Law, Religion, and Secular Order’ (2019) 7 Journal of Law, Religion and State 104.

11 By ‘critical secularism studies’, we mean a school of thought that critiques common presumptions about secularism and recognises that ‘religion’ is a modern, Western category. See Talal Asad, *Genealogies of Religion* (Johns Hopkins UP 1993); Gil Anidjar, ‘Secularism’ (2006) 33 Critical Inquiry 52; David Scott and Charles Hirschkind (eds), *Powers of the Secular Modern* (Stanford UP 2006).

12 Of course, this comparison of Islam to Judaism is not unique. As Robert Yelle elaborated about colonial India, ‘Christian attitudes toward Judaism served as a model for the interpretation of Hinduism and informed the marginalization of Hindu ritual laws’: Robert A Yelle, ‘The Hindu Moses: Christian Polemics against Jewish Ritual and the Secularization of Hindu Law under Colonialism’ (2009) 49 History of Religions 141, 147.

13 On conventional comparisons of Judaism and Islam, see Lena Salaymeh, ‘“Comparing” Jewish and Islamic Legal Traditions: Between Disciplinarity and Critical Historical Jurisprudence’ (2015) 2 Critical Analysis of Law, New Historical Jurisprudence 153.

14 We surveyed how secular law generates both Judaeophobia and Islamophobia in a forthcoming piece: Lena Salaymeh and Shai Lavi, ‘Secularism’ in Sol Goldberg, Scott Ury and Kalman Weiser (eds), *Key Concepts in the Study of Antisemitism* (Palgrave Macmillan forthcoming).

15 ‘Iceland’s Mooted Circumcision Ban Sparks Religious Outrage’ *BBC News* (19 February 2018) < www.bbc.com/news/world-europe-43111800> accessed 11 June 2020.

16 Rachael Revesz, ‘Norwegian Ruling Party Votes to Ban Circumcision for Men under 16 Years Old’ *The Independent* (London, 8 May 2017) <www.independent.co.uk/news/world/europe/norwegian-ruling-progress-party-ban-circumcision-men-under-16-years-old-vote-annual-conference-a7723746.html> accessed 11 June 2020.

17 Morten Frisch, ‘Denmark Doctors Declare Circumcision of Healthy Boys “Ethically Unacceptable”’ *Huffington Post* (12 January 2017) <www.huffingtonpost.com/entry/denmarks-29000-doctors-declare-circumcision-of-healthy_us_58753ec1e4b08052400ee6b3> accessed 11 June 2020.

18 Roberta Cowan, ‘Dutch Doctors Urge End to Male Circumcision’ *Reuters* (23 September 2011) <www.reuters.com/article/us-dutch-circumcision-health/dutch-doctors-urge-end-to-male-circumcision-idUSTRE78M3R620110923> accessed 11 June 2020.

19 On German Catholicism, see Ernst Wolfgang Böckenförde, *Der deutsche Katholozismus im Jahr 1933: Eine kritische Betrachtung* (Kosel-Verlag 1961); Mark Edward Ruff, *The Battle for the Catholic Past in Germany, 1945–1980* (CUP 2017).

20 There is a large body of scholarship on the historical situation of Jews in Germany and on the contemporary situation of Muslims in Germany; because these historical contexts do not bear directly on the model that we propose in this piece, we will not engage with this literature. On Jews, see eg Michael A Meyer and others (eds), *German-Jewish History in Modern Times* (Columbia UP 1996). On Muslims, see eg Ala Al-Hamarneh and Jörn Thielmann, *Islam and Muslims in Germany* (Brill 2014). For a general comparison of Jews and Muslims, see Shai Lavi, ‘Unequal Rites: Jews, Muslims and the History of Ritual Slaughter in Germany’ in Jose Brunner and Shai Lavi (eds), *Juden und Muslime in Deutschland: Recht, Religion, Identität* (Wallstein Verlag 2009).

21 See n 11.

22 Sarah Shortall, ‘Beyond a Christian Genealogy of the Secular’ *The Immanent Frame* (6 September 2017) <https://tif.ssrc.org/2017/09/06/history-and-theorizing-the-secular/#Shortall> accessed 11 June 2020.

23 Salaymeh and Lavi (n 14).

24 Lena Salaymeh, ‘Secular Translations of the Islamic Tradition: Converting Islamic Law into “Sharia” and Ethics’ Journal of Islamic Ethics (forthcoming).

25 By way of example, see Aaron R Petty, ‘Accommodating Religion’ (2015) 83 Tenn L Rev 529, 573; George C Freeman III, ‘The Misguided Search for the Constitutional Definition of Religion’ (1982) 71 Geo LJ 1519; Helen Yomtov Herman, ‘Comment: History and Utility of the Supreme Court’s Present Definition of Religion’ (1980) 26 Loy L Rev 87, 102; Courtney Miller, ‘Note: Spiritual but Not Religious: Rethinking the Legal Definition of Religion’ (2016) 102 Va L Rev 833, 846; Mark Strasser, ‘Free Exercise and the Definition of Religion: Confusion in the Federal Courts’ (2015) 53 Hous LR 909, 936.

26 Tim Jensen, ‘When is Religion, Religion, and a Knife, a Knife—and Who Decides?: The Case of Denmark’ in Taussig-Rubbo, Yelle and Sullivan (n 3) (Denmark’s presumably secular law applies a Protestant understanding of religion); Petty (n 25) 530 (‘reflects a Christian and largely Protestant worldview’); L Scott Smith, ‘Constitutional Meanings of Religion Past and Present: Explorations in Definition and Theory’ (2004) 14 Temple Political Civil Rights Law Review 89, 92.

27 Winnifred Fallers Sullivan, *The Impossibility of Religious Freedom* (Princeton UP 2005). See also Petty (n 25) 573; Freeman (n 25).

28 ECtHR Research Division, ‘Guide to Article 9: Freedom of Thought, Conscience, and Religion’ (2015) 7.

29 Daniel Philpott and Timothy Samuel Shah, ‘In Defense of Religious Freedom: New Critics of a Beleaguered Human Right’ (2016) 31 Journal of Law and Religion 380, 384.

30 ECtHR Research Division, ‘Overview of the Court’s Case-Law on Religious Freedom’ (2013) 9. See also Calo (n 4).

31 Saba Mahmood and Peter G Danchin, ‘Politics of Religious Freedom: Contested Genealogies’ (2014) 113 South Atlantic Quarterly 1, 5, emphasis in the original.

32 In recent years, at least two large research projects have focused on religious freedom from a critical secularism perspective: ‘Regulating Religion: Secularism and Religious Freedom in the Global Era’, comprised of Kari Telle (project leader), Michael Hertzberg and Christine Jacobsen (funded by FRISAM, Chr Michelsen Institute); and ‘Politics of Religious Freedom’, comprised of Saba Mahmood, Elizabeth Shakman Hurd, Winnifred Fallers Sullivan and Peter Danchin (funded by Henry R Luce Initiative on Religion and International Affairs).

33 Peter Danchin and others, ‘Politics of Religious Freedom: Case Studies’ (2014) 29 Maryland Journal of International Law 293, 304. See also Elizabeth Shakman Hurd and Winnifred Fallers Sullivan, ‘Symposium: Re-thinking Religious Freedom (Editors’ Introduction)’ (2014) 29 Journal of Law and Religion 358.

34 Elizabeth Shakman Hurd, *Beyond Religious Freedom: The New Global Politics of Religion* (Princeton UP 2015).

35 Samuel Moyn, ‘From Communist to Muslim: European Human Rights, the Cold War, and Religious Liberty’ (2014) 113 South Atlantic Quarterly 63.

36 Philpott and Shah (n 29) 390.

37 IDLO, *Freedom of Religion or Belief and the Law: Current Dilemmas and Lessons Learned* (2016) 7. Likewise, Mahmood and Danchin (n 4) 154 argue that ‘in all modern states we can see a consistent pattern of protecting state-sanctioned traditions or dominant religions and a corresponding insensitivity to and denial of the claims of minority, nontraditional, or unpopular religious groups’.

38 Moyn (n 35) 65 observes that ‘Already before 9/11 the European Court … treated Islam as a second-class religion not entitled to the same sort of consideration as the Christian faith. Since then, it has issued a series of decisions that grant European states wide latitude to ban Muslim symbols’.

39 Salaymeh and Lavi (n 14).

40 Philpott and Shah (n 29) 388.

41 Agrama (n 3) 502, emphasis in the original.

42 As previously noted, there are other dimensions of secularisation that are beyond the scope of this article. In addition, there are aspects of each node (religiosity, religious law and religious group) that we do not investigate; by way of example, religiosity includes a notion of irrationality that is contrasted with secularism’s purported ‘rationality’.

43 Berger noted that ‘law manifests a degree of comfort with religion as belief and displays a kind of anxiety and awkwardness with religion as practice. These respective reactions to belief and practice fit comfortably within the liberal framework of constitutional rights’: Benjamin L Berger, ‘Law’s Religion: Rendering Culture’ (2007) 45 Osgoode Hall LJ 277, 303.

44
*Beschneidung*, Landgericht Köln [Cologne District Court], Judgment of 7 May 2012, 151 Ns 169/11 (Ger).

45 For a comprehensive account of the case and its aftermath, see Hendrik Pekárek, ‘The Law on Circumcision in Germany’ in Elisha Ancselovits and George R Wilkes (eds), *Jewish Law and Academic Discipline: Contributions from Europe* (Deborach Charles Publications 2016). For a legal analysis, see Bijan Fateh-Moghadam, ‘Criminalizing Male Circumcision? Case Note: Landgericht Cologne, Judgment of 7 May 2012–No 151 Ns 169/11’ (2012) 13 German Law Journal 1131. See also Diana Aurenque and Urban Wiesing, ‘German Law on Circumcision and Its Debate: How an Ethical and Legal Issue Turned Political’ (2015) 29 Bioethics 203; David Abraham, ‘Circumcision: Immigration, Religion, History, and Constitutional Identity in Germany and the US’ (2017) 18 German Law Journal 1745; Stephen R Munzer, ‘Secularization, Anti-minority Sentiment, and Cultural Norms in the German Circumcision Controversy’ (2015) 37 University of Pennsylvania Journal of International Law 503.

46 Landgericht Köln [Cologne District Court], Judgment of 7 May 2012, 151 Ns 169/11 (Ger), reversing Amtsgericht Köln [Cologne Local Court], Judgment of 21 September 2011, 528 Ds 30/11 (Ger). For an English translation of the district court opinion, see Alexander Aumüller, ‘Unofficial Translation of 151 Ns 169/11’ (*Durham University*, 10 July 2012) <www.dur.ac.uk/ilm/newsarchive/?itemno=14984> accessed 9 June 2020.

47 ibid.

48 Elisa Oddone, ‘German Court Circumcision Ban Meets Wave of Criticism’ *Reuters* (28 June 2012) <www.reuters.com/article/us-germany-circumcision/german-court-circumcision-ban-meets-wave-of-criticism-idUSBRE85R1F020120628> accessed 11 June 2020.

49 ‘Circumcision Ruling Is “a Shameful Farce for Germany”’ *Spiegel Online* (13 July 2012) <www.spiegel.de/international/germany/german-press-review-on-outlash-against-court-s-circumcision-ruling-a-844271.html> accessed 11 June 2020.

50 Gareth Jones, ‘Circumcision Ban Makes Germany “Laughing Stock”: Merkel’ *Reuters* (17 July 2012) <www.reuters.com/article/us-germany-circumcision/circumcision-ban-makes-germany-laughing-stock-merkel-idUSBRE86G0EW20120717> accessed 11 June 2020.

51 Bundesgesetzblatt [Federal Law Gazette] Jahrgang 2012 Teil I Nr 61 (27 December 2012) 2749–50. The law is codified at §1631(d) of the Civil Code (BGB). See also Gerhard Robbers, ‘Recent Legal Developments in Germany: Infant Circumcision and Church Tax’ (2013) 15 Ecc LJ 69.

52 Fateh-Moghadam (n 45) 1139.

53 Munzer (n 45) 547.

54 ‘Mehrheit der Deutschen gegen Beschneidungsgesetz’ *Spiegel Online* (22 December 2012) <www.spiegel.de/politik/deutschland/studie-mehrheit-der-deutschen-gegen-beschneidungsgesetz-a-874473.html> accessed 11 June 2020. Munzer (n 45) 522 reports different polls that suggest Germans were rather evenly split on the issue of circumcision.

55 By way of example, see ‘Titelthema: Recht auf körperliche Unversehrtheit’ *Deutsche Hebammenzeitschrift* (18 January 2019) <https://hpd.de/artikel/titelthema-recht-koerperliche-unversehrtheit-16405> accessed 11 June 2020.

56 Wissenschaftlicher Dienst des Deutschen Bundestags, *Die zivil- und strafrechtliche Regelung der Beschneidung von Jungen im deutschen Recht: Zur Vereinbarkeit mit höherrangigem Recht* (2018) WD7-3000-211/18 <www.bundestag.de/resource/blob/592120/4b24671a655ea9fa0012bf6dfc22aa69/wd-7-211-18-pdf-data.pdf> accessed 12 June 2020.

57 Aumüller (n 46) 3.

58 Fateh-Moghadam (n 45) 1140 (‘the assumption of the court that male circumcision sort of irreversibly determines the religious affiliation of the child’).

59 For an attempt to justify this line of reasoning, see Kai Möller, ‘Ritual Male Circumcision and Parental Authority’ (2017) 8 Jurisprudence 461. The justification is somewhat paradoxical since one simultaneously assumes that religion is a matter of faith and that it can be inscribed irreversibly on the body.

60 Aumüller (n 46) 2.

61 Fateh-Moghadam (n 45) 1137 observes that parents usually have discretionary authority on ‘bodily interventions’ of their children.

62 Although German law began recognising a third gender in 2019, operations on babies for the purposes of gender binary conformity were common in the past; as with circumcision, these surgeries take place without consent of the child. See Claudia Wiesemann and others, ‘Ethical Principles and Recommendations for the Medical Management of Differences of Sex Development (DSD)/Intersex in Children and Adolescents’ (2010) 169 European Journal of Pediatrics 671, 672 (‘a moratorium on nonemergency intersex surgery in early childhood is not justified from an ethical point of view’).

63 In addition, there is a long colonial history of using consent to criticise and prohibit native practices. Dirks explains that ‘the focus on consent and agency worked to mask the coercion of colonial power itself, its capacity to define what is acceptable and what is not, what is civilized and what is not, and why it is that the extraordinary burden of knowledge and responsibility is arrogated by the colonizer’: Nicholas B Dirks, ‘The Policing of Tradition: Colonialism and Anthropology in Southern India’ (1997) 39 Comparative Studies in Society and History 182, 202.

64
*Babylonian Talmud*, Sanhedrin 44a. See Jacob Katz, ‘אוה לארשי אטחש יפ לע ףא (‘Though He Sinned, He Remains an Israelite’)’ (1958) Tarbiz 203.

65 Gal 5:2–6, NRSV. In Romans 9:30–32 (NRSV), Paul also emphasised belief over law or ritual practice: ‘^30^What then are we to say? Gentiles, who did not strive for righteousness, have attained it, that is, righteousness through faith; ^31^but Israel, who did strive for the righteousness that is based on the law, did not succeed in fulfilling that law. ^32^Why not? Because they did not strive for it on the basis of faith, but as if it were based on works. They have stumbled over the stumbling-stone.’

66 Abraham (n 45) 1759 observed that ‘one must wonder why so many German ethicists and jurists cannot even imagine infant baptism as violating any of the legal-constitutional strictures they see transgressed by circumcision. Secularism and liberal Protestantism are more closely tied than most advocates of either would care to acknowledge’.

67 For a discussion of Pauline Christianity’s ideas surrounding spiritual circumcision, see Lena Salaymeh, *The Beginnings of Islamic Law: Late Antique Islamicate Legal Traditions* (CUP 2016) ch 4.

68 William Galston, ‘Mark of Belonging: Why Circumcision is No Crime’ *Commonweal* (5 May 2014) <www.commonwealmagazine.org/mark-belonging> accessed 11 June 2020.

69 See generally Peter Richardson and Stephen Westerholm, *Law in Religious Communities in the Roman Period: The Debate over Torah and Nomos in Post-biblical Judaism and Early Christianity* (Wilfrid Laurier UP 1991).

70 Aumüller (n 46) 4.

71
*Palestinian Talmud*, Nedarim 3:9; *Midrash Tanhuma*, Tazria 19.

72 On circumcision in both the Jewish and Islamic traditions, see Salaymeh (n 67) ch 4. ‘Orthodox Muslims’ is not equivalent or comparable to ‘Orthodox Jews’, but instead refers to Muslims who adhere to orthodox Islam. For a definition of orthodoxy in the Islamic tradition, see Salaymeh (n 67) Introduction. We recognise that there are heterodox Muslims who do not practise circumcision.

73 Yelle (n 12) 143.

74 Benjamin L Berger, *Law’s Religion: Religious Difference and the Claims of Constitutionalism* (University of Toronto Press 2015). See also Berger (n 43).

75 ECtHR Research Division, ‘Overview of the Court’s Case-Law’ (n 30) 8.

76 ECtHR Research Division, ‘Guide to Article 9’ (n 28) 9.

77 Sullivan (n 27) 92.

78 Herman (n 25) 102; Miller (n 25) 846; Strasser (n 25) 936.

79 Asad (n 11) 47.

80 The Research Division of the ECtHR notes that ‘most of the rights recognised under Article 9 are individual rights … some of these rights may have a collective aspect’: ECtHR Research Division, ‘Overview of the Court’s Case-Law’ (n 30) 8. See also William P Marshall, ‘Religion as Ideas: Religion as Identity’ (1996) 7 Journal of Contemporary Legal Issues 385, 386.

81 By way of example, see the ECtHR’s discussion of religious groups acting ‘on behalf of its adherents’: ECtHR Research Division, ‘Guide to Article 9’ (n 28) 6.

82
*Valsamis v. Greece* (App no 21787/93) ECtHR 18 December 1996).

83 ECtHR Research Division, ‘Overview of the Court’s Case-Law’ (n 30) 17.

84 ECtHR Research Division, ‘Guide to Article 9’ (n 28) 21.

85 Marshall (n 80) 386. On religious choice, see generally James S Bielo, *Anthropology of Religion: The Basics* (Routledge 2015).

86 ECtHR Research Division, ‘Guide to Article 9’ (n 28) 24.

87
*Dogru v France* (App no 27058/05) ECtHR 4 December 2008; *Kervanci v France* (App no 31645/04) ECtHR 4 March 2009.For a summary of ECtHR cases on religious clothing, see ECtHR Press Unit, ‘Factsheet – Religious Symbols and Clothing’ (2016).

88 Anna A Korteweg and Gökce Yurdakul, *The Headscarf Debates: Conflicts of National Belonging* (Stanford UP 2014). On Islamophobia in public debates about headscarves, see Lena Salaymeh, ‘Imperialist Feminism and Islamic Law’ (2019) 17 Hawwa 333.

89 Aumüller (n 46) 3.

90 Najāshī ʿAlī Ibrāhīm, *Khiṣāl al-fiṭrah fī al-fiqh al-islāmī* (al-Taqadum 1980) 32.

91 See eg Muḥammad ibn Abī Bakr Ibn Qayyim al-Jawzīyah (d 1350; Syria), *Tuḥfat al-mawdūd bi-āḥkām al-mawlūd* (al-Maktabah al-Qayyimah 1977) 142.

92 Hendrik Pekárek, ‘Circumcision Indecision in Germany’ (2015) 4 Journal of Law, Religion and State 1, 6.

93 § 5 RelKErzG; Germann, in BeckOK GG (41st edn 15 May 2019) Art 4 GG n 27.

94 Munzer (n 45) 534.

95 Abraham (n 45) 1753.

96 Bundesgesetzblatt (n 51), which also provides a comprehensive analysis.

97 Ned Stafford, ‘German Ethics Council Backs Male Circumcision on Religious Grounds’ (2012) 345 British Medical Journal 4.

98 NRSV Bible. Other biblical sources for this prescribed day of circumcision are Genesis 17:12 (circumcision is at eight days old) and Genesis 21:4 (Isaac was eight days old when Abraham circumcised him).

99 Lavi (n 20).

100 See *Muhammad v Paruk* 553 F Supp 2d 893 (ED Mich). See also Adam Schwartzbaum, ‘The Niqab in the Courtroom: Protecting Free Exercise of Religion in a Post-Smith World’ (2011) 159 U Pa L Rev 1533; Pascale Fournier and Erica See, ‘The Naked Face of Secular Exclusion: Bill 94 and the Privatization of Belief’ (2012) 30 Windsor Yearbook of Access to Justice 63.

101 Richard Adams, ‘Senior Ofsted Official Backs Headteacher over Hijab Ban for Under Eights’ *The Guardian* (1 February 2018) <www.theguardian.com/education/2018/feb/01/ofsted-chief-backs-headteacher-over-hijab-ban-for-under-eigh> accessed 11 June 2020.

102 See generally Israel Jacob Yuval, *Two Nations in your Womb: Perceptions of Jews and Christians in Late Antiquity and the Middle Ages* (University of California Press 2008); Jacob Neusner, Ernest S Frerichs and Caroline McCracken-Flesher (eds), *‘To See Ourselves as Others See Us’: Christians, Jews, ‘Others’ in Late Antiquity* (Scholars Press 1985).

103 On Paul’s views of law, see Sigfred Pedersen, ‘Paul’s Understanding of the Biblical Law’ (2002) 44 Novum Testamentum 1.

104 The accusation that Judaism, in contradistinction to Christianity, is not a true religion has a long history dating back to early Christianity. It gained new traction in the post-Lutheran tradition and is central to modern German theology, philosophy and law. See Christian Wiese, *Wissenschaft des Judentums und protestantische Theologie im wilhelminischen Deutschland—Ein Schrei ins Leere?* (Mohr Siebeck 1999). On the relationship between Protestant theology and anti-Semitism, see Christopher J Probst, *Demonizing the Jews: Luther and the Protestant Church in Nazi Germany* (Indiana UP 2012).

105 Although a Protestant, Kant does not represent the entirety of the Protestant Christian tradition and is cited here only as an example.

106 Immanuel Kant, *Religion within the Bounds of Bare Reason* (first published 1793, JW Semple tr, Thomas Allen 1838), 165.

107 ibid 148–51.

108 On custom in Jewish and Islamic legal traditions, see Gideon Libson, ‘Halakhah and Reality in the Gaonic Period: Taqqanah, Minhag, Tradition and Consensus: Some Observations’ in Daniel Frank (ed), *The Jews of Medieval Islam: Community, Society, and Identity* (Brill 1995); Gideon Libson, ‘On the Development of custom as a source of law in Islamic law: Al-rujūʿu ilā al-ʿurfi aḥadu al-qawāʿidi al-khamsi allatī yatabannā ʿalayhā al-fiqhu’ (1997) 4 Islamic Law and Society 131.

109 On the socio-political process of differentiating between laws and norms (or ethics), see Salaymeh (n 24).

110
*Lautsi v Italy* App no 30814/06 (ECtHR, 18 March 2011).

111 Shehab Khan, ‘German State Orders All Government Buildings to Display a Christian Cross’ *The Independent* (London, 24 April 2018) <www.independent.co.uk/news/world/europe/germany-bavaria-christian-crosses-government-building-religion-a8320481.html> accessed 11 June 2020.

112 On Jewish and Islamic legal orthodoxy, see Salaymeh (n 67) ch 5.

113 On colonial codifications of Islamic law, see eg Scott Alan Kugle, ‘Framed, Blamed and Renamed: The Recasting of Islamic Jurisprudence in Colonial South Asia’ (2001) 35 Modern Asian Studies 257; David S Powers, ‘Orientalism, Colonialism, and Legal History: The Attack on Muslim Family Endowments in Algeria and India’ (1989) 31 Comparative Studies in Society and History 535.

114 Swathi Shanmugasundaram, ‘Anti-Sharia Law Bills in the United States’ (*Southern Poverty Law Center*, 5 February 2018) <www.splcenter.org/hatewatch/2018/02/05/anti-sharia-law-bills-united-states> accessed 11 June 2020. See also Lena Salaymeh, ‘Propaganda, Politics, and Profiteering: Islamic Law in the Contemporary US’ *Jadaliyya* (29 September 2014) <www.jadaliyya.com/pages/index/19408/propaganda-politics-and-profiteering_islamic-law-i> accessed 11 June 2020.

115 William T Cavanaugh, *The Myth of Religious Violence: Secular Ideology and the Roots of Modern Conflict* (OUP 2009).

116 J Steven Svoboda, Peter W Adler and Robert S Van Howe, ‘Circumcision is Unethical and Unlawful’ (2016) 44 Journal of Law, Medicine & Ethics 263, 265.

117 Kersten Knipp, ‘To Cut, or Not to Cut? The Never-Ending Debate around Circumcision’ *Deutsche Welle* (7 May 2017) <http://p.dw.com/p/2cZQW> accessed 11 June 2020.

118 Female circumcision is often depicted in the West as a ‘primitive’ African practice, but it is also frequently associated with Islam. On Islamophobic depictions of female circumcision, see Salaymeh (n 87).

119 Munzer (n 45) 511.

120 Munzer (n 45).

121 A similar consideration was explicitly made by the German Constitutional Court in the case of animal slaughter: Jews could not be denied the right to perform traditional practices of slaughter because the Nazis had banned the practice.

122 On the distinctions between ‘Jew’ and ‘Muslim’, as well as Jewish and Islamic traditions, see Lena Salaymeh, ‘Between Scholarship and Polemic in Judeo-Islamic Studies’ (2013) 24 Islam and Christian–Muslim Relations 407; Salaymeh (n 13); Lena Salaymeh, ‘Taxing Citizens: Socio-legal Constructions of Late Antique Muslim Identity’ (2016) 23 Islamic Law and Society 333.

123 For a more thorough elaboration of the distinctions between Jewish and Islamic traditions about circumcision, see Salaymeh (n 67) ch 4.

124 Genesis 17:9–12, NRSV (^9^God said to Abraham, ‘As for you, you shall keep my covenant, you and your offspring after you throughout their generations. ^10^This is my covenant, which you shall keep, between me and you and your offspring after you: Every male among you shall be circumcised. ^11^You shall circumcise the flesh of your foreskins, and it shall be a sign of the covenant between me and you. ^12^Throughout your generations every male among you shall be circumcised when he is eight days old’). See also *Babylonian Talmud*, Shabbat 137b. And see Lawrence A Hoffman, *Covenant of Blood: Circumcision and Gender in Rabbinic Judaism* (University of Chicago Press 1996).

125 The Babylonian Talmud reports that Beit Shammai (the dissenting opinion) ruled that a few drops of the ‘covenant blood’ must be drawn from the convert; in comparison, Beit Hillel (the normative opinion) ruled that this symbolic circumcision was unnecessary. *Babylonian Talmud*, Shabbat 135a. What is significant is that this debate even took place, since it points to the significance of blood in Jewish circumcision.

126 Nehal Bhuta, ‘Two Concepts of Religious Freedom in the European Court of Human Rights’ (2012) EUI Working Paper Law 2012/33 1, 2 < https://cadmus.eui.eu/bitstream/handle/1814/24678/LAW_2012_33_Bhuta_ReligiousFreedom.pdf?sequence=1&isAllowed=y> accessed 12 June 2020.

127 Bhuta (n 125).

128 Bhuta (n 125).

129 We elaborated the implications of the state’s classification of religious groups in Salaymeh and Lavi (n 14).

130 Cavanaugh (n 114) 4.

131 Brian Klug, ‘The Limits of Analogy: Comparing Islamophobia and Antisemitism’ (2014) 48 Patterns of Prejudice 442, 454; Nasar Meer and Tehseen Noorani, ‘A Sociological Comparison of anti-Semitism and anti-Muslim Sentiment in Britain’ (2008) 56 The Sociological Review 195, 209.

132 On terrorism as a product of modernity, see Mahmood Mamdani, *Good Muslim, Bad Muslim: America, the Cold War, and the Roots of Terror* (Three Leaves Press 2004).

133 Lavi (n 20).

134 For the specific example of how Islamic law is propagandised as a threat to the US public sphere, see Salaymeh (n 113). See also Elsadig Elsheikh, Basima Sisemore and Natalia Ramirez Lee, ‘Legalizing Othering: The United States of Islamophobia’ (2017) University of California, Berkeley Research Report.

135 Meer and Modood explain, ‘what is critical to the racialisation of a group is not the invocation of a biology but a radical “otherness” and the perception and treatment of individuals in terms of physical appearance and descent’: Nasar Meer and Tariq Modood, ‘For “Jewish” Read “Muslim”? Islamophobia as a Form of Racialisation of Ethno-religious Groups in Britain Today’ (2012) 1 Islamophobia Studies Journal 35, 39.

136 IDLO (n 37) 23.

137 On late antique Muslim identity as a form of citizenship, see Salaymeh, ‘Taxing Citizens’ (n 121).

138 Valerie Stoker, ‘Drawing the Line: Hasidic Jews, Eruvim, and the Public Space of Outremont, Quebec’ (2003) 43 History of Religions 18.

139 Davina Cooper, ‘Talmudic Territory – Space, Law, and Modernist Discourse’ (1996) 23 Journal of Law and Society 529; Charlotte Elisheva Fonrobert, ‘Installations of Jewish Law in Public Urban Space: An American ERUV Controversy’ (2015) 90 Chi-Kent L Rev 63.

140 Count Stanislas-Marie-Adélaide Clermont-Tonnerre, ‘Speech on Religious Minorities and Questionable Professions (1789)’ in Lynn Hunt (ed), *The French Revolution and Human Rights: A Brief Documentary History* (St Martin’s Press 1996).

141 Carl W Ernst, ‘Introduction’ in Carl W Ernst (ed), *Islamophobia in America: The Anatomy of Intolerance* (Palgrave Macmillan 2013) 4.

142 We thank Jakob Skovgaard-Petersen for informing us about this debate in Denmark.

143 James Renton and Ben Gidley, ‘Introduction: The Shared Story of Europe’s Ideas of the Muslim and the Jew – a Diachronic Framework’ in Ben Gidley and James Renton (eds), *Antisemitism and Islamophobia in Europe: A Shared Story?* (Palgrave Macmillan 2017) 5.

144 See eg Jonathan Fox and Yasemin Akbaba, ‘Securitization of Islam and Religious Discrimination: Religious Minorities in Western Democracies, 1990–2008’ (2015) 13 Comparative European Politics 175.

145 Salaymeh and Lavi (n 14).

146 On German Jewish physicians and their advocacy of male circumcision, see John M Efron, *Medicine and the German Jews: A History* (Yale UP 2001) ch 6.

147 See Robin E Judd, *Contested Rituals: Circumcision, Kosher Butchering and Jewish Political Life in Germany, 1848–1933* (Cornell Press 2007) 21.

148 Shaye JD Cohen, *Why Aren’t Jewish Women Circumcised? Gender and Covenant in Judaism* (University of California Press 2005) 184.

149 Herbert Auer, ‘Hayum Schwarz, der letzte Rabbiner in Hürben’ in Peter Fassl (ed), *Geschichte und Kultur der Juden in Schwaben II* (Jan Thorbecke Verlag 2000).

150 Leora F Batnitzky, *How Judaism Became a Religion: An Introduction to Modern Jewish Thought* (Princeton UP 2011).

151 Nadeem Elyas is discussed in Munzer (n 45) 526.

152 On the diversity of opinions and practices concerning Muslim circumcision, see Salaymeh (n 67) ch 4.

## Notes

We thank Peter Danchin, Yaacob Dweck, Rhiannon Graybill, participants in the University of Michigan Legal Theory Workshop, the 2018–2019 Davis Center Fellows (Princeton University) and the LAPA works-in-progress seminar (Princeton University) for providing feedback on this piece. We thank Philomena Hindermann for her research assistance.

