# Religion is Secularised Tradition: Jewish and Muslim Circumcisions in Germany

**DOI:** 10.1093/ojls/gqaa063

**Published:** 2021-01-12

**Authors:** Lena Salaymeh, Shai Lavi

(Oxford Journal of Legal Studies, Vol. 0, No. 0 (2020), pp. 1–28; doi: 10.1093/ojls/gqaa028)

In the originally published version of this manuscript, there was an error in First and Corresponding author Lena Salaymeh’s email address. The email address should read: lena.salaymeh@area.ox.ac.uk.

This error has now been corrected online.

